# Persistent D-dimer Elevation in a Yolk Sac Germ Cell Tumor Without Thrombosis: A Potential Surrogate Marker of Tumor Necrosis

**DOI:** 10.7759/cureus.86341

**Published:** 2025-06-19

**Authors:** Kasun Maduranga, C. M. D Selvarajah, Dilip C Madanayake, Sumudu Palihawadana, Eshantha Perera

**Affiliations:** 1 Department of Pulmonary Medicine, National Hospital for Respiratory Diseases, Welisara, LKA; 2 Department of Radiology, National Hospital for Respiratory Diseases, Welisara, LKA

**Keywords:** ct pulmonary angiogram, elevated d-dimer, germ cell tumors, pulmonary embolism (pe), yolk sac tumor

## Abstract

D-dimer is a widely used biomarker for thromboembolic events, but its role in oncology beyond clot detection remains underexplored. While transient elevation is common in cancer patients, persistent elevation without evidence of thrombosis is less well understood, particularly in germ cell tumors. We report the case of a 32-year-old male patient with a large anterior mediastinal yolk sac tumor who developed persistently elevated D-dimer levels despite multiple negative investigations for thrombosis, including CT pulmonary angiogram, ventilation-perfusion scan, and Doppler ultrasonography. Following debulking surgery, the patient showed clinical improvement and normalization of tumor markers, but D-dimer levels remained elevated at 2.4-2.5 mg/L. This case raises the possibility that persistent D-dimer elevation may reflect tumor necrosis or ongoing vascular remodeling rather than thrombosis or infection. If recognized, such a pattern could help clinicians avoid unnecessary anticoagulation and provide an adjunctive marker for residual tumor activity. D-dimer may have potential as a surrogate indicator of tumor dynamics in selected oncology settings. Further research is warranted to validate its prognostic or monitoring value in germ cell tumors.

## Introduction

D-dimer is a fibrin degradation product widely used as a diagnostic marker for thrombotic events, particularly deep vein thrombosis (DVT) and pulmonary embolism (PE). Elevated D-dimer levels indicate ongoing fibrinolysis and can be seen in various conditions, including thrombosis, disseminated intravascular coagulation (DIC), infections, trauma, and advanced malignancies. However, its role beyond thromboembolic disorders, especially in oncology, remains underexplored [1]. Emerging evidence suggests that elevated D-dimer levels in cancer patients may correlate with tumour burden and prognosis in certain malignancies, including lung and gastrointestinal cancers [2,3].

D-dimer elevation is often attributed to a hypercoagulable state in patients with malignancies, but it is not traditionally recognized as a serum tumor marker. In germ cell tumors (GCTs), standard tumor markers include alpha-fetoprotein (AFP), beta-human chorionic gonadotropin (β-hCG), and lactate dehydrogenase (LDH), which assist in diagnosis, staging, and monitoring treatment response [4]. The clinical relevance of persistently elevated D-dimer levels in the absence of thrombosis or infection in GCT patients is unclear.

We present the case of a 32-year-old male patient with a history of yolk sac germ cell tumor who exhibited persistently elevated D-dimer levels despite the absence of radiologically confirmed thromboembolic disease, active infection, or coagulopathy. This case raises the possibility that D-dimer may reflect ongoing tumor necrosis or remodeling and highlights its potential utility as a surrogate marker of tumor activity in selected oncological contexts. 

## Case presentation

A 32-year-old previously healthy Sri Lankan man was transferred to the National Hospital for Respiratory Disease, Welisara, for evaluation of a right-sided pleural effusion. His initial symptoms included a five-day history of low-grade fever, anorexia, and vomiting. He denied any history of tuberculosis or significant past illness.

On examination, he appeared cachectic, with a weight of 41 kg and a height of 154 cm (BMI 17.3 kg/m²). His vital signs were stable: blood pressure of 133/78 mmHg, heart rate of 88 beats per minute, and respiratory rate of 20 breaths per minute. Chest auscultation revealed diminished breath sounds and dullness to percussion over the right hemithorax, consistent with a moderate effusion. Abdominal and neurological examinations were unremarkable.

Initial laboratory investigations showed elevated inflammatory markers, with no evidence of bacterial or mycobacterial infection (Table 1). A chest radiograph demonstrated consolidation in the right lower zone with a moderate pleural effusion. 

**Table 1:** Laboratory investigation summary.

Test	Patient Value	Reference Range	Units
Erythrocyte sedimentation rate	95	<20	mm/h
C-reactive protien	68	<5	mg/L
Procalcitonin	Negative	<0.1	ng/mL
Blood culture	No growth	Sterile	-
Sputum culture	No growth	Sterile	-
Mantoux test	No induration	<10	mm
GeneXpert (tuberculosis)	Negative	Negative	-
Pleural fluid – adenosine deaminase	9.7	<40	U/L
Pleural fluid – LDH	1323	<200	U/L
D-dimer (initial)	2.4	<0.5	mg/L
D-dimer (48-hour repeat)	2.5	<0.5	mg/L
D-dimer (3 weeks postoperative)	2.4	<0.5	mg/L
Beta-human chorionic gonadotropin	167.5	<5	mIU/mL
Alpha-fetoprotein	2.3	<10	ng/mL
Liver function tests	Normal	Normal	-
Coagulation profile	Normal	Normal	-
Serum immunoglobulins	Normal	Normal	-
ECG	Normal	Normal	-
Transthoracic echocardiogram	Normal	Normal	-

**Figure 1:**
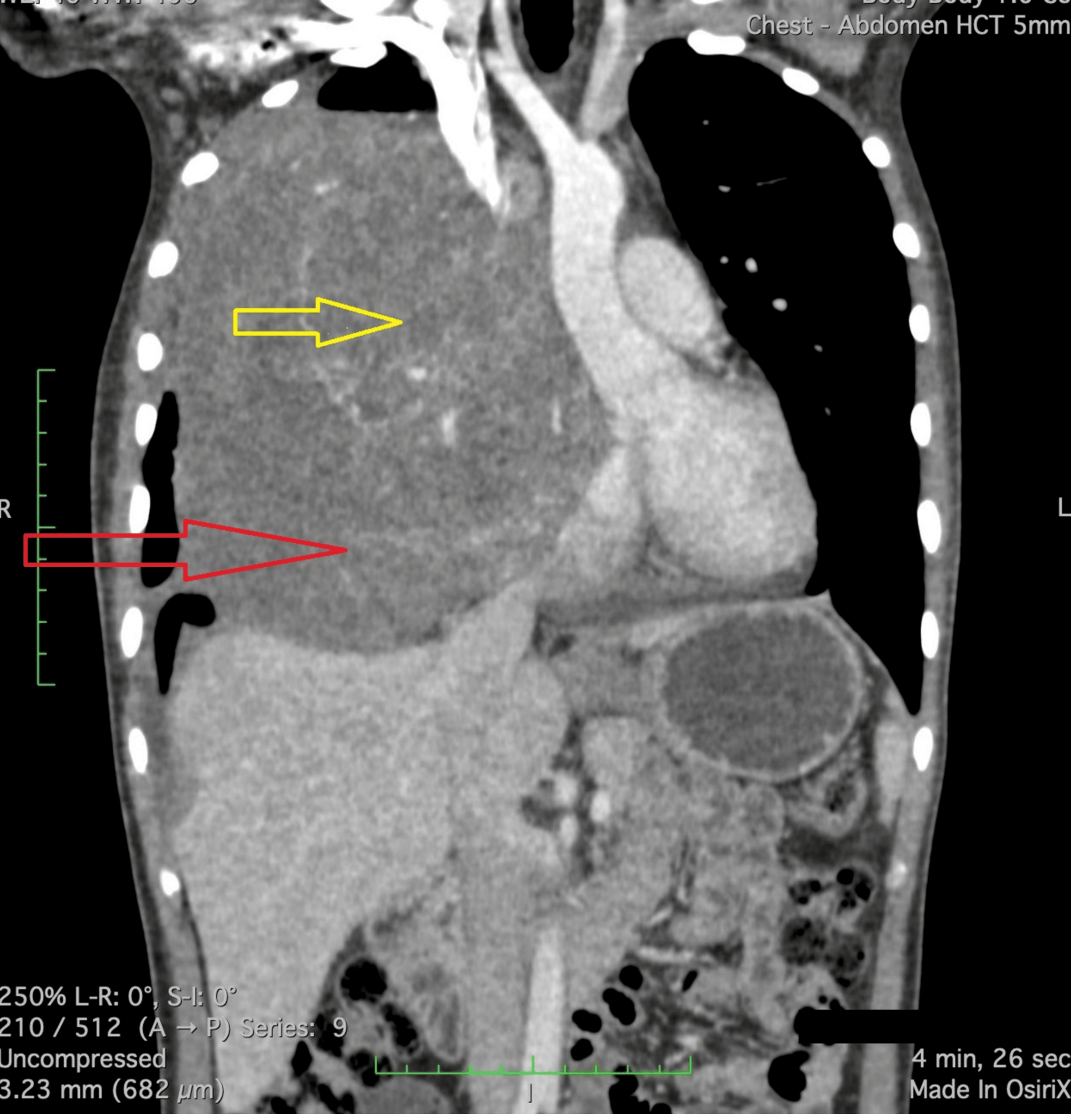
CECT Chest showing anterior mediastinal mass. Coronal CT image of the chest demonstrating a heterogeneously enhancing anterior mediastinal mass (red arrow) with a central, non-enhancing cystic or necrotic area (yellow arrow). The mass displaces adjacent structures without clear evidence of invasion. These features are consistent with a germ cell tumour, likely a yolk sac variant. CECT: contrast-enhanced computed tomography

Diagnostic thoracentesis revealed an exudative effusion. Contrast-enhanced computed tomography (CECT) of the chest identified a large, heterogeneously enhancing anterior mediastinal mass measuring 11.1 cm (anteroposterior) × 13 cm (transverse) × 15.5 cm (craniocaudal) with bulky mediastinal lymphadenopathy and right pleural effusion (Figure 1). No evidence of pulmonary embolism was seen.

Ultrasound-guided core-needle biopsy of the mass revealed features consistent with a yolk sac tumor. A testicular ultrasound was unremarkable.

**Figure 2:**
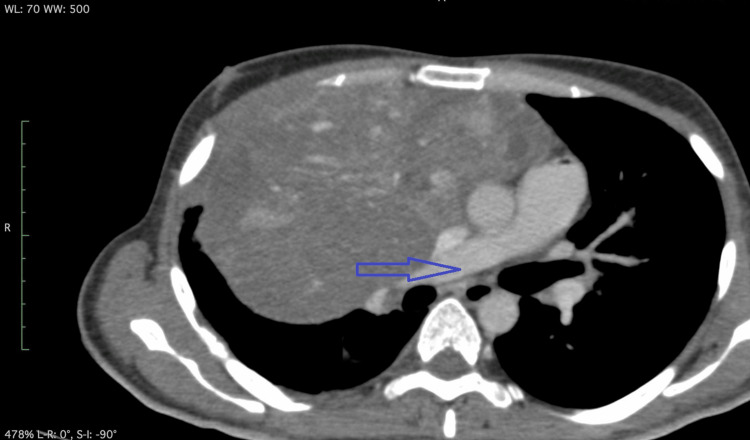
CTPA showing no evidence of thromboembolism. Axial image from CTPA demonstrating the main pulmonary artery (blue arrow) with no filling defects. indicating the absence of pulmonary embolism. CTPA: computed tomography pulmonary angiogram

Serum tumor markers showed normal alpha-fetoprotein (AFP) and elevated beta-human chorionic gonadotropin (β-hCG). While awaiting surgical intervention, the patient developed an acute onset of dyspnea, accompanied by tachycardia and mild desaturation (SpO2 90% on room air), though he remained hemodynamically stable. The electrocardiogram (ECG) was normal. A markedly elevated D-dimer level prompted further evaluation with a CT pulmonary angiogram (Figure 2), Tc-99m MAA perfusion scan, and lower limb Doppler ultrasound, all of which ruled out thromboembolic disease. Repeat D-dimer testing after 48 hours remained persistently elevated. Additional evaluation, including procalcitonin, coagulation profile, liver function tests, and echocardiography, was all within normal limits.

**Figure 3:**
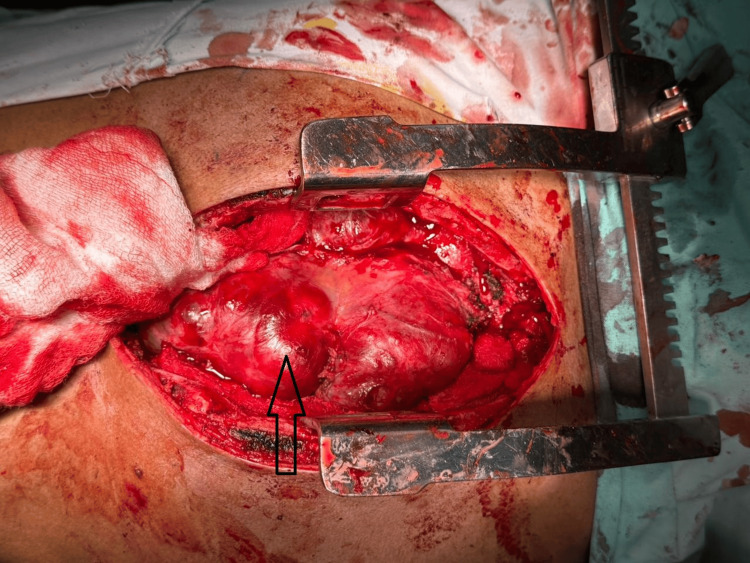
Intraoperative view of mediastinal tumor debulking. Intraoperative photograph taken during median sternotomy shows a large, firm, reddish anterior mediastinal mass (black arrow) being exposed with the aid of a rib retractor. The mass arises from the mediastinum and appears encapsulated with surface irregularities. Approximately 60% of the tumor was resected during this procedure.

The patient subsequently underwent median sternotomy and debulking of the mediastinal tumor (Figure 3), with approximately 60% of the tumor mass resected. Postoperatively, the patient showed symptomatic and radiological improvement, reducing pleural effusion and inflammatory markers. β-hCG levels also declined. However, D-dimer levels remained persistently elevated with a repeat level of 2.4 mg/L measured three weeks after surgery, despite clinical stability and resolution of inflammatory parameters.

## Discussion

D-dimer is a widely used biomarker in the evaluation of suspected thromboembolic disease. Elevated levels typically reflect increased fibrin formation and breakdown, commonly seen in conditions such as deep vein thrombosis, pulmonary embolism, DIC, infection, and malignancy [5]. While cancer itself is a well-recognized prothrombotic state, the interpretation of elevated D-dimer levels in the absence of thrombosis or infection remains poorly defined, particularly in the context of germ cell tumors.

In our case, the patient had persistently elevated D-dimer levels ranging from 2.3 to 5.0 mg/L, including a value of 2.4 mg/L, three weeks after surgery, despite repeated negative evaluations for thromboembolic events. CTPA, ventilation-perfusion scanning, and lower limb Doppler ultrasound failed to demonstrate thrombosis. Negative procalcitonin and cultures excluded infectious causes. The patient was clinically stable and did not demonstrate signs of systemic inflammation or coagulopathy.

The mediastinal mass was confirmed to be a yolk sac germ cell tumor, and histopathological examination of the resected specimen showed extensive necrosis with areas of hemorrhage, but no viable tumor cells. After debulking surgery, the patient’s clinical symptoms improved and tumor markers normalized, yet D-dimer levels remained elevated. This raised the possibility that tumor necrosis, vascular remodeling, or ongoing microscopic prothrombotic activity might contribute to persistent fibrin turnover.

While elevated D-dimer is not considered a standard tumor marker in germ cell tumors, some reports suggest a correlation between tumor burden, necrosis, and fibrinolytic activity [6]. In other solid tumors, such as pancreatic or gastric cancers, D-dimer levels have been shown to reflect disease progression, correlate with necrotic tumor components, and sometimes decline after complete resection [7]. Although no definitive studies exist for germ cell tumors, our case raises the hypothesis that D-dimer could be a surrogate indicator of ongoing tumor remodeling, especially in the absence of thrombosis or infection.

Several mechanisms may explain this phenomenon [8]. Tumor necrosis can lead to the release of tissue factor and other procoagulant microparticles, which may trigger local or subclinical coagulation activation followed by fibrinolysis. Additionally, large mediastinal masses may exert mechanical pressure on adjacent vasculature, causing endothelial injury and further contributing to a prothrombotic environment. In this case, the persistent elevation of D-dimer despite tumor debulking suggests that microscopic disease or vascular remodeling may continue to stimulate fibrinolytic pathways.

Importantly, distinguishing between thrombotic and non-thrombotic causes of elevated D-dimer is critical to avoid unnecessary anticoagulation, particularly in oncology patients. This case highlights the need for careful clinical correlation and multidisciplinary evaluation when interpreting persistently elevated D-dimer levels.

To our knowledge, this is one of the first reports suggesting a potential role for D-dimer as a surrogate marker of residual tumor necrosis or activity in germ cell tumors. Further studies are needed to validate this observation and explore whether serial D-dimer measurements could provide additional insight into treatment response or early recurrence in selected oncologic settings.

## Conclusions

This case highlights a rare but clinically significant observation of persistently elevated D-dimer levels in a patient with a yolk sac germ cell tumor without thrombosis or infection. Despite surgical debulking and clinical improvement, D-dimer levels remain high, suggesting a potential link to tumor necrosis or ongoing vascular remodeling. While D-dimer is not traditionally recognized as a tumor marker, this case suggests the potential utility of D-dimer as a non-invasive surrogate indicator of residual tumor activity or response to therapy. Recognizing this pattern may help clinicians avoid unnecessary anticoagulation and prompt further investigation into the underlying disease process. Prospective studies are warranted to clarify the utility of D-dimer as a potential biomarker in germ cell tumors and other malignancies
